# Melatonin Enhances Mitophagy by Upregulating Expression of Heat Shock 70 kDa Protein 1L in Human Mesenchymal Stem Cells under Oxidative Stress

**DOI:** 10.3390/ijms20184545

**Published:** 2019-09-13

**Authors:** Yeo Min Yoon, Hyung Joo Kim, Jun Hee Lee, Sang Hun Lee

**Affiliations:** 1 Medical Science Research Institute, Soonchunhyang University Seoul Hospital, Seoul 336-745, Koreahyungjoothomaskim@gmail.com (H.J.K.); j-school@hanmail.net (J.H.L.); 2 Departments of Biochemistry, Soonchunhyang University College of Medicine, Cheonan 330-930, Korea

**Keywords:** oxidative stress, mesenchymal stem cells, mitochondria, melatonin, mitophagy, HSPA1L, parkin

## Abstract

Human mesenchymal stem cells (hMSCs) are a potent source of cell-based regenerative therapeutics used to treat patients with ischemic disease. However, disease-induced oxidative stress disrupts mitochondrial homeostasis in transplanted hMSCs, resulting in hMSC apoptosis and reducing their efficacy post-transplantation. To address this issue, we evaluated the effects of melatonin on cellular defense mechanisms and mitophagy in hMSCs subjected to oxidative stress. H_2_O_2_-induced oxidative stress increases the levels of reactive oxygen species and reduces membrane potential in hMSCs, leading to mitochondrial dysfunction and cell death. Oxidative stress also decreases the expression of 70-kDa heat shock protein 1L (HSPA1L), a molecular chaperone that assists in the recruitment of parkin to the autophagosomal mitochondrial membrane. Decreased expression of HSPA1L destabilizes parkin, thereby impairing mitophagy. Our results indicate that treating hMSCs with melatonin significantly inhibited mitochondrial dysfunction induced by oxidative stress, which decreased hMSCs apoptosis. In damaged hMSCs, treatment with melatonin increased the levels of HSPA1L, which bound to parkin. The interaction between HSPA1L and parkin increased membrane potential and levels of oxidative phosphorylation, resulting in enhanced mitophagy. Our results indicate that melatonin increased the expression of HSPA1L, thereby upregulating mitophagy and prolonging cell survival under conditions of oxidative stress. In this study, we have shown that melatonin, a readily available compound, can be used to improve hMSC-based therapies for patients with pathologic conditions involving oxidative stress.

## 1. Introduction

Human mesenchymal stem cells (hMSCs), which are present mainly in the bone marrow, adipose tissue, dental pulp, and the umbilical cord, can potentially be used in cell-based therapies and regenerative medicine because of their continued self-renewal, multidirectional differentiation, and immunomodulatory abilities [1,2,3]. In our previous study, we demonstrated that MSC-based therapy is a promising modality for targeting ischemic disease associated with chronic kidney dysfunction [4]. However, hMSCs show low therapeutic efficacy when utilized in cell-based therapies; this is because hMSCs lose viability when subjected to oxidative stress that arises in various pathophysiological conditions such as inflammation and ischemia [5,6,7]. Sustained exposure of hMSCs to oxidative stress induces disruption in their mitochondrial membrane potential and results in mitochondrial dysfunction [5,6,7,8]. Because mitochondria play a pivotal role in cell metabolism, damaged mitochondria need to be eliminated in a timely manner to recover cellular homeostasis and function. Failure to eliminate damaged mitochondria results in apoptosis, which is the primary cause of failure in hMSC-based therapies [9,10].

Mitochondria play an important role in ATP synthesis during aerobic respiration. In this process, reactive oxygen species (ROS), such as the superoxide anion (O_2_^−^), hydroxyl radicals (OH^−^), hydroperoxyl (HO_2_^−^), and hydrogen peroxide (H_2_O_2_), are formed as metabolites of mitochondrial oxidative phosphorylation [9,11,12]. Under conditions of oxidative stress, mitochondria can synthesize ROS at a level that extensively disrupts mitochondrial homeostasis, altering the composition of lipids, proteins, and nucleic acids. The resulting disruption in the composition of the mitochondrial membrane impairs electrochemical potential of the membrane, leading to mitochondrial dysfunction and cell death [11,13,14]. Because mitochondrial damage can lead to cell death, timely elimination of excess ROS is important for cell survival [15,16]. Elevated levels of ROS trigger mitophagy, a cellular process in which lysosomes selectively scavenge for, and eliminate, damaged mitochondria. [11,17,18]. Under conditions of oxidative stress, mitophagy is upregulated to prevent the accumulation of dysfunctional mitochondria. Therefore, regulation of mitophagy in hMSCs is important for enhancing the viability of hMSCs transplanted into ischemic injury sites.

Melatonin (N-acetyl-5-methoxytryptamine) is synthesized from serotonin and is the main hormone secreted by the pineal gland at night under normal light/dark conditions. It was first discovered and isolated from the bovine pineal gland by Aaron Lerner [19]. Since then, this methoxyindole compound has been found in various tissues such as bone marrow, ovary, testes, gut, placenta and liver [20,21,22]. Previous studies have shown that melatonin-treated MSCs can facilitate therapeutically functional recovery in myocardial infarction, skin wounds, lung ischemia-reperfusion injury, and sepsis-induced kidney injury [23,24,25]. In our previous study, we showed that melatonin-treated hMSCs increase neovascularization in hind limb ischemia by augmenting the activity of mitophagy-mediated processes [25]. A recent study showed that the levels of 70-kDa heat shock protein 1L (HSPA1L) are increased under oxidative and metabolic stress [26]. In initial steps of mitophagy, HSPA1L (a member of the 70-kD heat shock protein (HSP70) family) transfers and tethers parkin to membranes of dysfunctional mitochondria, thereby inducing mitophagy [27,28]. In this study, we examined whether enhanced interactions between HSPA1L and parkin underlie melatonin-enhanced mitophagy and resulting reduction in oxidative stress.

## 2. Results

### 2.1. Oxidative Stress Impairs Mitochondrial Function in hMSCs

**Figure 1:**
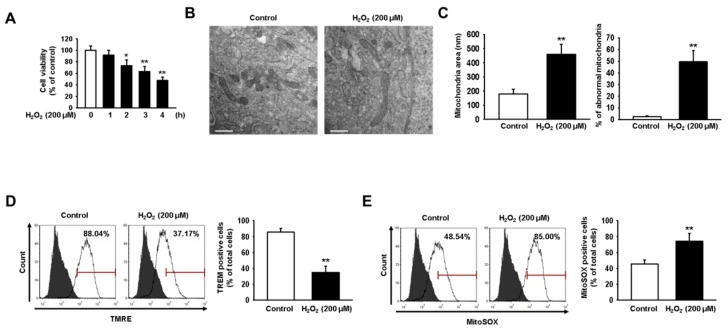
H_2_O_2_-induced oxidative stress reduces human mesenchymal stem cell (hMSC) viability by inducing mitochondrial dysfunction. (**A**) MTT assay was used to assess the viability of hMSCs treated with 200 μM H_2_O_2_ and that of untreated hMSCs. Values represent the mean ± SEM. * *p* < 0.05 or ** *p* < 0.01 vs. control. (**B**) Transmission electron microscopy (TEM) was used to evaluate mitochondrial morphology in hMSCs treated for 24 h with H_2_O_2_ (200 μM) and in untreated hMSCs. Scale bar = 500 nm. (**C**) Quantitative analyses of morphometric data and percentages of abnormal mitochondria showing swelling and severely disrupted cristae. Images were obtained using TEM. Values represent the mean ± SEM. ** *p* < 0.01 vs. control. (**D**) Tetramethylrhodamine, ethyl ester (TMRE)-positive hMSCs, with and without treatment using H_2_O_2_ (200 μM), were quantified via fluorescence-activated cell sorting (FACS). Values represent the mean ± SEM. ** *p* < 0.01 vs. control. (**E**) MitoSOX-positive hMSCs with and without treatment using H_2_O_2_ (200 μM) were quantified via FACS. Values represent the mean ± SEM. ** *p* < 0.01 vs. control.

In our previous study, significant oxidative stress conditions were induced when hMSCs were treated with 200 μM H_2_O_2_ for 4 h [5]. To confirm that oxidative stress decreases the viability of hMSCs, we exposed hMSCs to 200 μM H_2_O_2_ for 0, 1, 2, 3, and 4 h (Figure 1A). Our results indicate that the viability of hMSCs was reduced by approximately 50% at 4 h post-treatment with H_2_O_2_. Therefore, in this study, we used treatment with 200 μM H_2_O_2_ to induce oxidative stress in hMSCs. Dysfunctional mitochondria were observed using transmission electron microscopy (TEM). Mitochondrial dysfunction was indicated by abnormalities in mitochondrial size and structure, while accumulation was evidenced by increased numbers of mitochondria (Figure 1B,C). Our findings show that oxidative stress caused a significant accumulation of impaired mitochondria in hMSCs treated with 200 μM H_2_O_2_ for 4 h. In addition, oxidative stress decreased mitochondrial membrane potential and increased the levels of mitochondrial ROS, as assessed using tetramethylrhodamine, ethyl ester (TMRE) and MitoSOX assays, respectively (Figure 1D,E). These results show that oxidative stress deteriorates mitochondrial function and impairs cellular homeostasis in hMSCs.

### 2.2. Melatonin-Treated hMSCs Show Increased HSPA1L Expression and Parkin Stability under Conditions of Oxidative Stress

**Figure 2:**
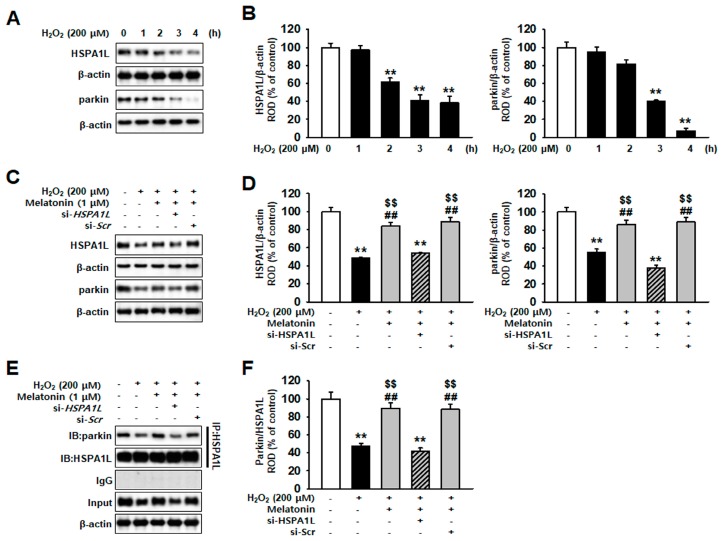
Melatonin enhances parkin stability by increasing HSPA1L expression in hMSCs subjected to oxidative stress. (**A**) Expression of HSPA1L and parkin in hMSCs treated with or without H_2_O_2_ for various time periods (0, 1, 2, 3, and 4 h). (**B**) The expression levels of HSPA1L and parkin were normalized with respect to that of β-actin. Values represent the mean ± SEM. ** *p* < 0.01 vs. untreated hMSCs. (**C**) Expression of HSPA1L and parkin in hMSCs pretreated with *HSPA1L* siRNA (si-*HSPA1L*) and 1 μM melatonin for 24 h. (**D**) Expression levels of HSPA1L and parkin were normalized relative to those of β-actin. Values represent the mean ± SEM. ** *p* < 0.01 vs. untreated hMSCs; ## *p* < 0.01 vs. H_2_O_2_-treated hMSCs; $$ *p* < 0.01 vs. melatonin-treated hMSCs pretreated with si-*HSPA1L*. (**E**) Immunoprecipitates with anti-HSPA1L were analyzed after treatment of melatonin-pretreated hMSCs with si-*HSPA1L* by western blot using an antibody that recognized parkin. (**F**) The levels of parkin bound to HSPA1L were normalized with respect to that of β-actin. Values represent the mean ± SEM. ** *p* < 0.01 vs. untreated hMSCs; ## *p* < 0.01 vs. melatonin-treated hMSCs; $$ *p* < 0.01 vs. melatonin-treated hMSCs pretreated with si-*HSPA1L*.

Translocation of parkin to the mitochondria is induced by the binding of parkin and HSPA1L; this is followed by initiation of mitophagy, which eliminates dysfunctional mitochondria [29]. The results obtained in this study indicate that expression of HSPA1L and parkin decreased time-dependently in hMSCs treated with H_2_O_2_ (200 μM) (Figure 2A,B). In addition, we observed reduced expression of HSPA1L in hMSCs that accumulated dysfunctional mitochondria. Our results demonstrate that hMSCs treated with melatonin showed augmented resistance against oxidative stress-induced apoptosis. We detected that treatment of hMSCs with melatonin increased the expression of HSPA1L and parkin (Figure S1A). In addition, pre-incubating hMSCs with melatonin restored the expression of HSPA1L and parkin, increasing their resistance to oxidative stress (Figure 2C,D). We also analyzed that the knockdown of HSPA1L in hMSCs results in the loss of melatonin function on parkin (Figure S3A,B and Figure 2C,D). To confirm that parkin stability was reduced by decreased expression of HSPA1L, we used immunoprecipitation (IP) to assess the level of binding between parkin and HSPA1L in hMSCs treated with melatonin (Figure 2E,F). Our results indicate that this effect was not present in HSPA1L-knockdown hMSCs (Figure 2E,F).

### 2.3. Mitophagy Pathway Is Enhanced via Increased Expression of HSPA1L in Melatonin-Treated hMSCs Subjected to Oxidative Stress

**Figure 3:**
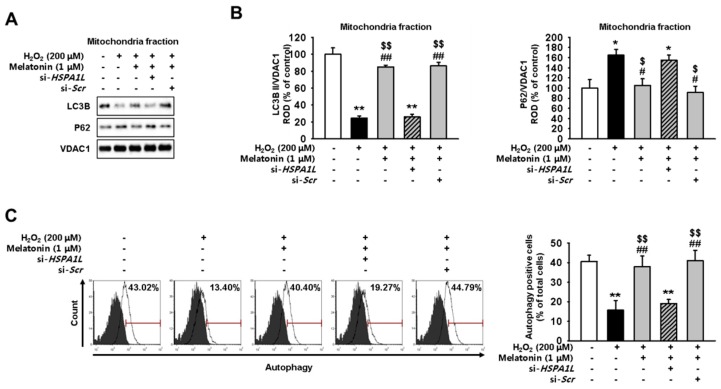
Treatment with melatonin enhances mitophagy by stabilizing parkin in hMSCs subjected to oxidative stress. (**A**) Expression of P62 and LC3B II in mitochondrial fractions obtained from melatonin-treated (1 μM, 24 h) hMSCs, si-*HSPA1L*-treated hMSCs, and untreated control hMSCs; all cell groups were subjected to oxidative stress. (**B**) Expression levels were normalized relative to those of VDAC1. Values represent the mean ± SEM. * *p* < 0.05, and ** *p* < 0.01 vs. untreated hMSCs; # *p* < 0.05, and ## *p* < 0.01 vs. H_2_O_2_-treated hMSCs; $ *p* < 0.05, and $$ *p* < 0.01 vs. melatonin-treated hMSCs pretreated with si-*HSPA1L*. (**C**) hMSCs treated with melatonin (1 μM, 24 h) and si-*HSPA1L* were quantified as autophagy-positive or autophagy-negative using FACS. Values represent the mean ± SEM. ** *p* < 0.01 vs. untreated hMSCs; ## *p* < 0.01 vs. melatonin-treated hMSCs; $$ *p* < 0.01 vs. melatonin-treated hMSCs pretreated with si-*HSPA1L*.

Next, we evaluated whether treatment with melatonin enhances mitophagy in hMSCs, thereby aiding in elimination of dysfunctional mitochondria. We confirmed that treatment of hMSCs with melatonin decreased the level of P62 and increased the expression of LC3B II in mitochondria (Figure S1B). For this, we assessed the expression levels of mitophagy-associated-proteins in mitochondrial fractions of melatonin-treated and untreated hMSCs subjected to oxidative stress. Our results, obtained using western blotting, show increased levels of LC3B II and decreased levels of P62 in hMSCs treated with melatonin and subjected to oxidative stress compared with the levels of untreated controls placed under oxidative stress (Figure 3A,B). Flow cytometric analysis indicated that pre-incubating hMSCs with melatonin increased the formation of autophagosome under conditions of oxidative stress (Figure 3C). Knockdown of HSPA1L in hMSCs decreased the expression of LC3B II and increased that of P62; these events attenuated the effects of melatonin and destabilized the HSPA1L/parkin interactions. These results indicate that melatonin promoted the mitophagy pathway via upregulation of HSPA1L/parkin axis in hMSCs placed under oxidative stress.

### 2.4. Melatonin-Treated hMSCs Show Enhanced Mitochondrial Function under Conditions of Oxidative Stress

**Figure 4:**
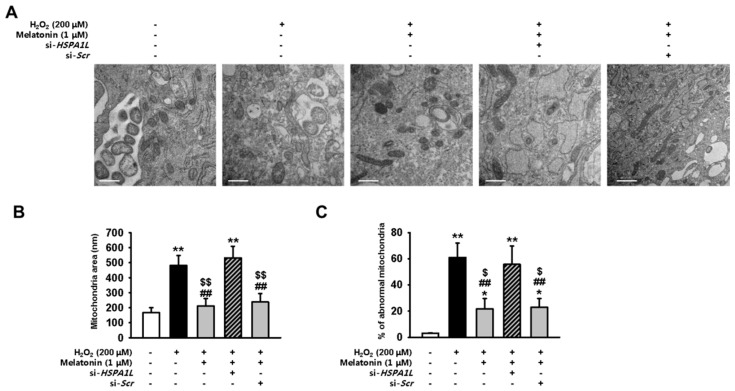
Melatonin increases the numbers of healthy mitochondria by stabilizing parkin in hMSCs subjected to oxidative stress. (**A**) Mitochondrial morphology was evaluated using TEM in hMSCs treated with melatonin (1 μM, 24 h) and si-*HSPA1L*, and subjected to oxidative stress. Scale bar = 500 nm. (**B**,**C**) Quantitative analyses of morphometric data, and percentages of abnormal mitochondria showing swelling and severely disrupted cristae, as evaluated using TEM. Values represent the mean ± SEM. * *p* < 0.05, and ** *p* < 0.01 vs. untreated hMSCs; ## *p* < 0.01 vs. melatonin–treated hMSCs; $ *p* < 0.05, and $$ *p* < 0.01 vs. melatonin-treated hMSCs pretreated with si-*HSPA1L*.

**Figure 5:**
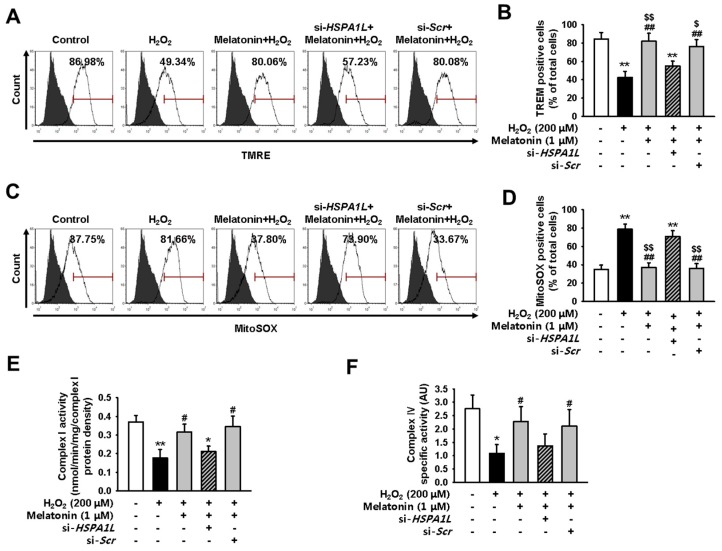
Melatonin enhances mitochondrial function by stabilizing parkin in hMSCs subjected to oxidative stress. (**A**) hMSCs were pre-treated with melatonin (1 μM, 24 h) and si-*HSPA1L*, and subjected to oxidative stress. Untreated hMSCs were subjected to oxidative stress only. All groups were assayed using TMRE, and TMRE-positive hMSCs were quantified using FACS. (**B**) Values represent the mean ± SEM. ** *p* < 0.01 vs. untreated hMSCs, ## *p* < 0.01 vs. H_2_O_2_-treated hMSCs, $ *p* < 0.05, and $$ *p* < 0.01 vs. melatonin-treated hMSCs pretreated with si-*HSPA1L*. (**C**) hMSCs were pre-treated with melatonin (1 μM, 24 h) and si-*HSPA1L*, and subjected to oxidative stress. Untreated hMSCs were subjected to oxidative stress only. All groups were assayed using MitoSOX, and MitoSOX-positive hMSCs were quantified using FACS. (**D**) Values represent the mean ± SEM. ** *p* < 0.01 vs. untreated hMSCs, ## *p* < 0.01 vs. H_2_O_2_-treated hMSCs, $$p < 0.01 vs. melatonin-treated hMSCs pretreated with si-*HSPA1L*. (**E** and **F**) Activity of complexes I (E) and IV (F) in hMSCs treated with melatonin (1 μM, 24 h) and si-*HSPA1L*. Values represent the mean ± SEM. * *p* < 0.05, and ** *p* < 0.01 vs. untreated hMSCs; # *p* < 0.05 vs. H_2_O_2_-treated hMSCs; $ *p* < 0.05 vs. melatonin-treated hMSCs pretreated with si-*HSPA1L*.

We next aimed to show that melatonin attenuates the effects of dysfunctional mitochondrial accumulation by upregulating the expression of HSPA1L in hMSCs subjected to oxidative stress. For this, we used TEM to evaluate changes in mitochondrial morphology in hMSCs pre-treated with melatonin and placed under conditions of oxidative stress. Pre-treatment with melatonin restored homeostatic mitochondrial size and decreased the number of dysfunctional mitochondria (Figure 4A–C). hMSCs pre-treated with melatonin also showed restored mitochondrial membrane potential and reduced levels of mitochondrial ROS under conditions of oxidative stress (Figure S4A and Figure 5A–D). To evaluate the function of electron transport chain (ETC), we measured the activities of ETC complexes I and IV in untreated hMSCs, and in those treated with melatonin, under conditions of oxidative stress. hMSCs treated with melatonin showed enhanced activities of ETC complexes I and IV under conditions of oxidative stress (Figure 5E,F). To further confirm that melatonin augments mitophagy via the HSPA1L/parkin axis, we evaluated whether HSPA1L-knockdown hMSCs would retain dysfunctional mitochondrial morphology under conditions of oxidative stress even after pre-treatment with melatonin (Figure 4A–C). Our results indicate that knockdown of HSPA1L in hMSCs disrupted mitochondrial membrane potential and increased the synthesis of mitochondrial ROS under conditions of oxidative stress; this activity was not dependent on treatment with melatonin (Figure 5A–D). Additionally, HSPA1L knockdown decreased the activities of ETC complexes I and IV in hMSCs subjected to oxidative stress; this activity was also not dependent on treatment with melatonin (Figure 5E,F). These data indicate that melatonin enhanced the mitophagy pathway by augmenting interactions between HSPA1L and parkin in hMSCs placed under oxidative stress.

### 2.5. Melatonin Protects hMSCs against Oxidative Stress by Augmenting Mitophagy

**Figure 6:**
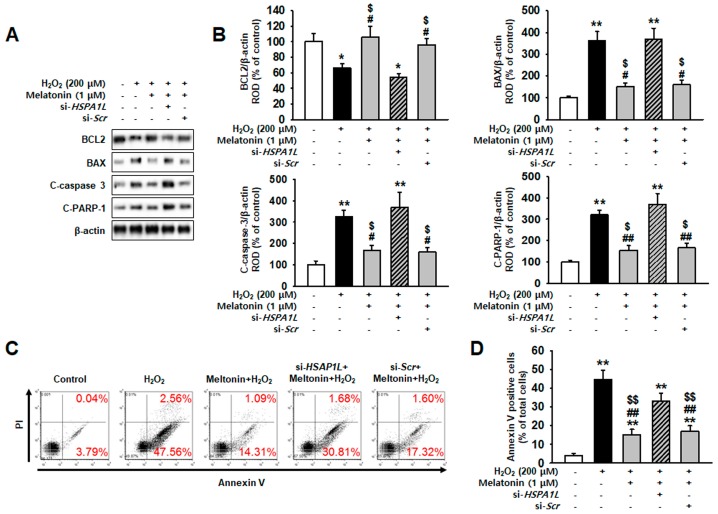
Melatonin protects against oxidative stress by preserving the stability of parkin. (**A**) Expression of anti-apoptotic protein BCL2, and pro-apoptotic proteins BAX, C-caspase-3, and C-PARP-1, in hMSCs treated with melatonin (1 μM, 24 h) and *HSPA1L* siRNA (si-*HSPA1L*) (**B**) Expression levels were normalized relative to those of β-actin. Values represent the mean ± SEM. * *p* < 0.05, and ** *p* < 0.01 vs. untreated hMSCs, # *p* < 0.05, and ## *p* < 0.01 vs. H_2_O_2_-treated hMSCs, $ *p* < 0.05 vs. melatonin-treated hMSCs pretreated with si-*HSPA1L*. (**C**) hMSCs were pre-treated with melatonin (1 μM, 24 h) and si-*HSPA1L*, and subjected to oxidative stress. Untreated hMSCs were subjected to oxidative stress only. (**D**) All groups were assayed using PI-Annexin V, and Annexin V-positive hMSCs were quantified using FACS. Values represent the mean ± SEM. ** *p* < 0.01 vs. untreated hMSCs, ## *p* < 0.01 vs. melatonin-treated hMSCs, $$ *p* < 0.01 vs. melatonin-treated hMSCs pretreated with si-*HSPA1L*.

To confirm that melatonin exerts anti-apoptotic effects and protects hMSCs against oxidative stress, we measured the expressions of anti-apoptotic protein BCL2 and pro-apoptotic proteins BAX, cleaved caspase-3, and cleaved PARP-1. Under conditions of oxidative stress, melatonin-treated hMSCs showed the increased expression of BCL2 and the decreased expressions of BAX, cleaved caspase 3, and cleaved PARP-1 (Figure 6A,B). Flow-cytometric analysis using annexin V showed that treatment with melatonin significantly decreased hMSCs apoptosis (Figure S5A and Figure 6C). However, knockdown of HSPA1L in hMSCs abolished the anti-apoptotic effects exerted by melatonin under oxidative stress (Figure 6A–C). These results indicate that melatonin upregulated mitophagy by increasing the expression of HSPA1L and inducing parkin stabilization, which eliminates the dysfunctional mitochondria.

## 3. Discussion

Various studies have revealed that the excessive levels of ROS under ischemic conditions cause MSCs to undergo extensive apoptosis, which decreases their effectiveness in the treatment [4,30,31,32]. To improve the therapeutic potential of MSCs in ischemic diseases, MSCs should acquire the resistance against ROS-mediated oxidative stress, which restrict MSC-mediated tissue regeneration and vascularization [33]. It is well known that melatonin enhances the efficacy of MSCs-based therapy in ischemic injuries such as skin wounds, focal cerebral ischemia, liver ischemic-reperfusion injury, and acute lung ischemia-reperfusion injury [34,35,36,37]. In addition, applications of MSCs for regenerative medicine in clinical trials (www.clinicaltrials.gov) have shown therapeutic effects in several diseases, including aplastic anemia, myocardial infarction, ulcerative colitis, chronic kidney diseases, liver cirrhosis, graft versus host disease, acute lung injury, and type I diabetes [38]. However, the underlying mechanisms of the melatonin effects are still unclear. In this study, we found that the treatment of MSCs with melatonin augmented cell viability by maintaining mitochondrial homeostasis under oxidative stress. Our results reveal that the positive effects of melatonin were associated with the expression of HSPA1L, which enhances mitophagy response that clears damaged mitochondria subjected to oxidative stress.

Mitochondria are a widely recognized source of ROS in animal cells [39]. Although ROS are normal metabolites of oxidative phosphorylation, uncontrolled accumulation of ROS induces significant damage to the very machinery that produces these ROS. Oxidative stress disrupts mitochondrial membrane potential, electron transport chain machinery, the fusion–fission cycle that determines the functional morphology, and mitophagy. [9,40,41]. The heightened levels of mitochondrial ROS cause mitochondrial DNA mutations and disruptions in the membrane compartments [9,40,41]. The resulting ROS-mediated uncoupling of the electron transport chain further induces distortions in mitochondrial morphology through elongation and fusion [42,43]. In addition, the irregular morphology inhibits the mitophagy probes from sensing the damaged mitochondria, hence the accumulation of dysfunctional mitochondria [44]. We showed that ROS-induced oxidative stress conferred the mitochondrial homeostatic imbalance with the accumulation of mitochondrial ROS. Dysfunctional mitochondria led to enhanced ROS formation, which, in turn, exacerbated mitochondrial health and further intensified oxidative stress in a self-perpetuating vicious cycle until apoptotic cell death [45,46].

**Figure 7:**
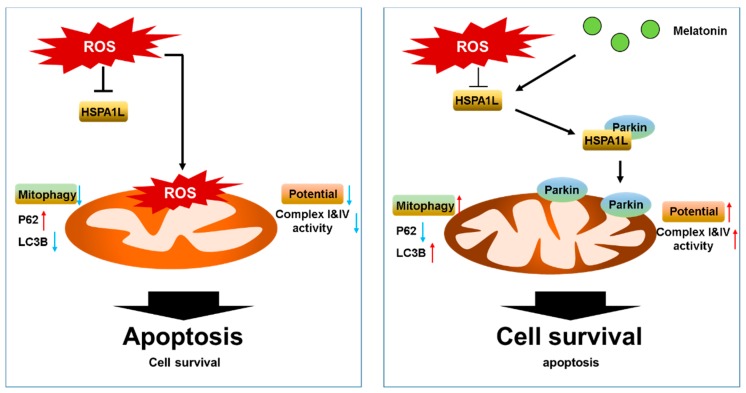
Schematic shows melatonin-induced resistance to oxidative stress (left), and how treatment with melatonin restored mitochondrial function by increasing the expression of HSPA1L and stabilizing parkin in hMSCs subjected to oxidative stress. Oxidative stress induces mitochondrial dysfunction by decreasing mitophagy, which results in accumulation of abnormal mitochondria. Melatonin-treated hMSCs increase their expression of HSPA1L, which stabilizes parkin-induced mitophagy and helps eliminate dysfunctional mitochondria; this cascade also increases mitochondrial membrane potential and decreases apoptosis in hMSCs placed under oxidative stress.

HSPA1L is a member of the HSP70 family. HSP70 proteins play important roles in protein quality control by assisting in the refolding of denatured proteins, preventing aggregation, and aiding in intracellular protein transport [47,48]. HSP70 is an important homeostatic regulator that reduces ROS levels and helps retain mitochondrial function during the inflammatory response [47,48]. Specifically, HSPA1L activates mitophagy by binding and translocating parkin to the damaged mitochondria [28]. Parkin is a cytoplasmic protein that initiates the mitophagy signaling cascade when damaged mitochondria are sensed. It is recruited to the mitochondrial outer membrane and interacts with a mitochondrial kinase PINK1, a mitochondrial protein that acts as a sensor of mitochondrial damage [49,50]. Parkin and PINK1 coordinate ubiquitination, proteasomal activation, and mitophagy response that may attenuate cell death [51]. In this study, we found that the treatment of melatonin with hMSCs increased the expressions of HSPA1L and parkin as well as their interaction under oxidative stress. Aligning with the previous findings, the enhanced HSPA1L/parkin interaction stimulated mitochondrial homeostasis from the oxidative stress-induced imbalance by increasing mitophagy activity, as indicated by the increased expression of LC3B and the reduced expression of p62 under western blot analysis [28]. Our results show that melatonin-dependent enhancement of mitophagy significantly decreased the numbers of dysfunctional mitochondria, restored mitochondrial morphology, suppressed the accumulation of mitochondrial ROS, and rescued mitochondrial membrane potential and ETC machinery. Thus, consistent with our hypothesis, apoptotic cell death was suppressed by the treatment of melatonin. However, the knockdown of HSPA1L reversed the effects of melatonin, suggesting that the upregulation of HSPA1L/parkin binding is a mechanism of melatonin-mediated antioxidative action (Figure 7).

To the best of our knowledge, this study is the first to demonstrate that the treatment with melatonin increases the expression of HSPA1L. To further reveal the underlying mechanisms on the relationship between HSPA1L and parkin, a future study needs to investigate the effect of overexpression of HSPA1L in MSCs under physiological and pathophysiological conditions. Our results reveal that melatonin can enhance mitophagy, thereby rescuing mitochondrial homeostasis and protecting hMSCs against the adverse effects of an oxidative stress environment. Therefore, our study suggests that melatonin is a potential adjuvant drug that enhances the efficacy of the MSC-based therapy for patients who suffer from ischemia.

## 4. Materials and Methods

### 4.1. Human MSC Cultures

Human adipose tissue-derived MSCs (hMSCs) were obtained from the American Type Culture Collection (Manassas, VA, USA). The hMSCs were confirmed negative for hepatitis B virus, hepatitis C virus, human immunodeficiency virus, syphilis, and mycoplasma. hMSCs expressed CD73 and CD 105 surface markers (Figure S2A) and showed adipogenic and osteogenic differentiation potentials when cultured with specific differentiation media [52]. hMSCs were cultured in alpha-Minimum Essential Medium (α-MEM; Gibco BRL, Gaithersburg, MD, USA) supplemented with 10% (*v*/*v*) fetal bovine serum (FBS; Gibco BRL) and 100 U/mL penicillin/streptomycin (Gibco BRL). hMSCs were maintained in a humidified incubator at 37 °C and 5% CO_2_.

### 4.2. Treatments Administered to hMSCs

hMSCs were washed twice with phosphate buffered saline (PBS), and then fresh α-MEM media supplemented with 10% FBS was added. To examine the apoptotic signaling pathway or mitophagy pathway, hMSCs (passage 4) were incubated with melatonin (1 μM) at 37 °C for 24 h and then treated with H_2_O_2_ (200 μM) for 0, 1, 2, 3, or 4  h.

### 4.3. Inhibition of HSPA1L Expression by RNA Interference

hMSCs (2  ×  10^5^) were seeded into 60-mm dishes and transfected with siRNA in serum-free Opti-MEM (Gibco BRL) using Lipofectamine 2000 (Thermo Fisher Scientific, Waltham, MA, USA) per manufacturer’s instructions. At 48 h post-transfection, total protein was extracted, and gene expression was determined using western blotting. siRNA used to target HSPA1L and a scrambled sequence were synthesized by Dharmacon (Lafayette, CO, USA).

### 4.4. Electron Microscopy

hMSCs were fixed in 3% glutaraldehyde and 2% paraformaldehyde in 0.1 M sodium cacodylate buffer at pH 7.3. Morphometric analyses (used to assess mitochondrial size and number of mitochondria per cell) were performed using ImageJ software (NIH; version 1.43). At least 10 cells per each low-magnification image (×10,000) were used to count the number of mitochondria per each hMSCs (identified by the presence of lamellar bodies). At least 100–150 individual mitochondria from each cell group were examined at high magnification (×25,000 and ×50,000) and used to assess mitochondrial perimeter and area.

### 4.5. Measurement of Mitochondrial Membrane Potential

To quantify the formation of mitochondrial O_2_^•−^, we used TMRE (Abcam, Cambridge, UK) to measure generation of mitochondrial superoxide in hMSCs subjected to oxidative stress. For this, hMSCs were trypsinized for 5 min, centrifuged at 1200 rpm for 3 min, washed with PBS twice, and incubated with 200 nM TMRE solution in PBS at 37 °C for 15 min. The cells were then washed two times with PBS and resuspended in 500 μL PBS. Following this, TMRE signaling was detected by fluorescence-activated cell sorting (FACS; Sysmex, Kobe, Japan). Cellular forward-scatter levels for TMRE-positive cells (number of events: 10^4^ cells) were analyzed using Flowing Software (DeNovo Software, Los Angeles, CA, USA).

### 4.6. Measurement of Mitochondrial Superoxide (O_2_^•−^) Generation

Generation of mitochondrial O_2_^•−^ in hMSCs was assayed using MitoSOX (Thermo Fisher Scientific). The cells in each group were trypsinized and centrifuged at 600 g for 5 min. The samples were then washed with PBS and incubated with 10 μM MitoSOX solution in phosphate-buffered saline (PBS) at 37 °C for 15 min. Next, the cells were resuspended in 500 μL PBS, and the total number of cells labeled using MitoSOX was measured via FACS (Sysmex, Kobe, Japan). MitoSOX-positive cells (number of events: 10^4^) were identified and analyzed using Flowing Software (DeNovo Software, Los Angeles, CA, USA).

### 4.7. Autophagy Assessment

Autophagosome-emitted fluorescence in hMSCs was evaluated using an Autophagy Assay Kit (Abcam). The cells were washed twice using PBS, then 100 μL detection reagent was added to each plate of hMSCs, and the cells were allowed to incubate for 30 min 37 °C. hMSCs were then washed with PBS and fixed using 4% formaldehyde for 20 min. The labeled hMSCs (number of events: 10^4^) were analyzed using FACS (Partec, Münster, Germany)

### 4.8. PI/Annexin V Flow Cytometric Analysis

hMSCs (number of events: 10^4^) apoptosis was assessed by labeling the cells with Annexin V-FITC and propidium iodide (PI) (De Novo Software, Los Angeles, CA, USA). Signal detection was performed using Cyflow Cube 8 (Partec, Münster, Germany), and data were analyzed using standard FSC Express (De Novo Software, Los Angeles, CA, USA).

### 4.9. Western Blotting

Total homogenates (20 μg protein) were separated using 8–12% sodium dodecyl sulfate-polyacrylamide gel electrophoresis (SDS-PAGE), and proteins were transferred to nitrocellulose membranes. The blots were washed with TBST (10 mM Tris-HCl (pH 7.6), 150 mM NaCl, and 0.05% Tween-20), blocked with 5% skim milk for 1 h, and incubated with the appropriate primary antibodies overnight at 4 °C using the dilutions recommended by the supplier. Antibodies specific for HSPA1L, parkin, LC3B, P62, and VDAC1 were purchased from Novus Biological (dilution of 1:1000; Centennial, CO, USA); those specific for BCL2, BAX, cleaved caspase 3, cleaved PARP-1, and β-actin were purchased from Santa Cruz Biotechnology (dilution of 1:500; Dallas, TX, USA). After incubation with primary antibodies, membranes were washed and incubated with goat anti-rabbit IgG (dilution of 1:5000) or goat anti-mouse IgG (dilution of 1:10,000) secondary antibodies conjugated to horseradish peroxidase (Santa Cruz Biotechnology) for 1 h at 25 °C. The bands were then visualized by enhanced chemiluminescence (Amersham Pharmacia Biotech, England, UK).

### 4.10. Immunoprecipitation

hMSCs were lysed using lysis buffer (1% Triton X-100 in 50 mM Tris-HCl (pH 7.4) containing 150 mM NaCl, 5 mM EDTA, 2 mM Na_3_VO_4_, 2.5 mM Na_4_PO_7_, 100 mM NaF, and protease inhibitors). Cell lysates (300  μg) were incubated with anti-HSPA1L antibody (Novus Biologicals) for 4 h, mixed with Protein A/G PLUS-Agarose Immunoprecipitation Reagent (Santa Cruz Biotechnology), and then incubated for an additional 12 h. The beads were then washed four times, and bound protein was released from the beads by boiling in SDS-PAGE sample buffer for 5 min. The precipitated proteins were analyzed by western blotting using anti-parkin primary antibody (Santa Cruz Biotechnology).

### 4.11. Evaluation of Electron Transport Chain Complex I Activity

Complex I activity was measured using a Complex I Enzyme Activity Assay kit (Abcam) following the manufacturer’s instructions. Briefly, 125–1250 µg/mL whole cell lysate was added to each well of a 96-well microplate, and the plate was incubated for 3 h at 25 °C. Each well was then washed three times. The mixture was diluted with dilution buffer to yield 20× nicotinamide adenine dinucleotide hydrogen (NADH) and 100× dye. The mixture was carefully added to each well (200 µL per well). Absorbance was measured immediately at 450 nm every minute for 30 min using an ELISA microplate reader (BMG Labtech, Ortenberg, Germany). Raw data were expressed as rate (mOD/min) per µg/mL of cell lysate.

### 4.12. Evaluation of Electron Transport Chain Complex IV Activity

Complex IV activity was measured using a Complex IV Enzyme Activity Assay kit (Abcam) following the manufacturer’s instructions. Briefly, 5 mg/mL of each sample was added to wells of a microplate and incubated for 3 h at 25 °C. The enzyme present in each well was immobilized by the bound monoclonal antibody. Each well was washed three times using potassium phosphate buffer. The solution was removed and replaced with 200 µL of assay solution containing potassium phosphate buffer and cytochrome complex (cyt c). Absorbance was measured every 1 to 5 min for 2 h at 550 nm using an ELISA microplate reader (BMG Labtech). Complex IV activity was calculated as (absorbance at time 1−absorbance at time 2)/Δt (min). The initial rate was decreased due to inhibition of complex IV reaction; therefore, the activity rate was always expressed as the initial rate of oxidation of cyt c.

### 4.13. Statistical Analysis

Results were expressed as mean ± standard error of the mean (SEM). Significant differences between groups were assessed using two-tailed Student’s *t*-test, or one- or two-way analysis of variance (ANOVA). Three or more groups were compared using Dunnett’s or Tukey’s post-hoc test. Data were considered significantly different at *p* < 0.05.

## Supplementary Materials

Supplementary materials can be found at https://www.mdpi.com/1422-0067/20/18/4545/s1.

## References

[1] Phinney D.G., Pittenger M.F. (2017). Concise review: Msc-derived exosomes for cell-free therapy. Stem Cells.

[2] Bian S., Zhang L., Duan L., Wang X., Min Y., Yu H. (2014). Extracellular vesicles derived from human bone marrow mesenchymal stem cells promote angiogenesis in a rat myocardial infarction model. J. Mol. Med..

[3] Teng X., Chen L., Chen W., Yang J., Yang Z., Shen Z. (2015). Mesenchymal stem cell-derived exosomes improve the microenvironment of infarcted myocardium contributing to angiogenesis and anti-inflammation. Cell. Physiol. Biochem. Int. J. Exp. Cell. Physiol. Biochem. Pharmacol..

[4] Yoon Y.M., Kim S., Han Y.S., Yun C.W., Lee J.H., Noh H., Lee S.H. (2019). Tudca-treated chronic kidney disease-derived hmscs improve therapeutic efficacy in ischemic disease via prp(c). Redox Biol..

[5] Yoon Y.M., Lee J.H., Yun S.P., Han Y.S., Yun C.W., Lee H.J., Noh H., Lee S.J., Han H.J., Lee S.H. (2016). Tauroursodeoxycholic acid reduces er stress by regulating of akt-dependent cellular prion protein. Sci. Rep..

[6] Yun S.P., Yoon Y.M., Lee J.H., Kook M., Han Y.S., Jung S.K., Lee S.H. (2018). Tauroursodeoxycholic acid protects against the effects of p-cresol-induced reactive oxygen species via the expression of cellular prion protein. Int. J. Mol. Sci..

[7] Han Y.S., Lee J.H., Jung J.S., Noh H., Baek M.J., Ryu J.M., Yoon Y.M., Han H.J., Lee S.H. (2015). Fucoidan protects mesenchymal stem cells against oxidative stress and enhances vascular regeneration in a murine hindlimb ischemia model. Int. J. Cardiol..

[8] Yoon Y.M., Lee J.H., Yun C.W., Lee S.H. (2019). Pioglitazone improves the function of human mesenchymal stem cells in chronic kidney disease patients. Int. J. Mol. Sci..

[9] Ott M., Gogvadze V., Orrenius S., Zhivotovsky B. (2007). Mitochondria, oxidative stress and cell death. Apoptosis Int. J. Program. Cell Death.

[10] Ott M., Robertson J.D., Gogvadze V., Zhivotovsky B., Orrenius S. (2002). Cytochrome c release from mitochondria proceeds by a two-step process. Proc. Natl. Acad. Sci. USA.

[11] Fan P., Xie X.H., Chen C.H., Peng X., Zhang P., Yang C., Wang Y.T. (2019). Molecular regulation mechanisms and interactions between reactive oxygen species and mitophagy. DNA Cell Biol..

[12] Rahal A., Kumar A., Singh V., Yadav B., Tiwari R., Chakraborty S., Dhama K. (2014). Oxidative stress, prooxidants, and antioxidants: The interplay. BioMed Res. Int..

[13] Sergi D., Naumovski N., Heilbronn L.K., Abeywardena M., O’Callaghan N., Lionetti L., Luscombe-Marsh N. (2019). Mitochondrial (dys)function and insulin resistance: From pathophysiological molecular mechanisms to the impact of diet. Front. Physiol..

[14] Thannickal V.J., Fanburg B.L. (2000). Reactive oxygen species in cell signaling. Am. J. Physiol. Lung Cell. Mol. Physiol..

[15] Truban D., Hou X., Caulfield T.R., Fiesel F.C., Springer W. (2017). Pink1, parkin, and mitochondrial quality control: What can we learn about parkinson’s disease pathobiology?. J. Park. Dis..

[16] Ashrafi G., Schwarz T.L. (2013). The pathways of mitophagy for quality control and clearance of mitochondria. Cell Death Differ..

[17] Narendra D., Kane L.A., Hauser D.N., Fearnley I.M., Youle R.J. (2010). P62/sqstm1 is required for parkin-induced mitochondrial clustering but not mitophagy; vdac1 is dispensable for both. Autophagy.

[18] MacVicar T. (2013). Mitophagy. Essays Biochem..

[19] Lerner A.B., Case J.D., Takahashi Y., Lee T.H., Mori W. (1958). Isolation of melatonin, the pineal gland factor that lightens melanocytes. J. Am. Chem. Soc..

[20] Reiter R.J., Rosales-Corral S., Tan D.X., Jou M.J., Galano A., Xu B. (2017). Melatonin as a mitochondria-targeted antioxidant: One of evolution’s best ideas. Cell. Mol. Life Sci. CMLS.

[21] Lee J.H., Yoon Y.M., Han Y.S., Jung S.K., Lee S.H. (2019). Melatonin protects mesenchymal stem cells from autophagy-mediated death under ischaemic er-stress conditions by increasing prion protein expression. Cell Prolif..

[22] Han Y.S., Kim S.M., Lee J.H., Jung S.K., Noh H., Lee S.H. (2019). Melatonin protects chronic kidney disease mesenchymal stem cells against senescence via prp(c)—dependent enhancement of the mitochondrial function. J. Pineal Res..

[23] Lee J.H., Han Y.S., Lee S.H. (2017). Potentiation of biological effects of mesenchymal stem cells in ischemic conditions by melatonin via upregulation of cellular prion protein expression. J. Pineal Res..

[24] Lee J.H., Yoon Y.M., Han Y.S., Yun C.W., Lee S.H. (2018). Melatonin promotes apoptosis of oxaliplatin-resistant colorectal cancer cells through inhibition of cellular prion protein. Anticancer Res..

[25] Han Y.S., Kim S.M., Lee J.H., Lee S.H. (2018). Co-administration of melatonin effectively enhances the therapeutic effects of pioglitazone on mesenchymal stem cells undergoing indoxyl sulfate-induced senescence through modulation of cellular prion protein expression. Int. J. Mol. Sci..

[26] Li G., Yang J., Yang C., Zhu M., Jin Y., McNutt M.A., Yin Y. (2018). Ptenalpha regulates mitophagy and maintains mitochondrial quality control. Autophagy.

[27] Weber H., Huhns S., Jonas L., Sparmann G., Bastian M., Schuff-Werner P. (2007). Hydrogen peroxide-induced activation of defense mechanisms against oxidative stress in rat pancreatic acinar ar42j cells. Free Radic. Biol. Med..

[28] Hasson S.A., Kane L.A., Yamano K., Huang C.H., Sliter D.A., Buehler E., Wang C., Heman-Ackah S.M., Hessa T., Guha R. (2013). High-content genome-wide rnai screens identify regulators of parkin upstream of mitophagy. Nature.

[29] Matsuda N., Sato S., Shiba K., Okatsu K., Saisho K., Gautier C.A., Sou Y.S., Saiki S., Kawajiri S., Sato F. (2010). Pink1 stabilized by mitochondrial depolarization recruits parkin to damaged mitochondria and activates latent parkin for mitophagy. J. Cell Biol..

[30] Colmegna I., Stochaj U. (2018). Msc—targets for atherosclerosis therapy. Aging.

[31] Bari E., Ferrarotti I., Torre M.L., Corsico A.G., Perteghella S. (2019). Mesenchymal stem/stromal cell secretome for lung regeneration: The long way through “pharmaceuticalization” for the best formulation. J. Control. Release Off. J. Control. Release Soc..

[32] Soria-Juan B., Escacena N., Capilla-Gonzalez V., Aguilera Y., Llanos L., Tejedo J.R., Bedoya F.J., Juan V., De la Cuesta A., Ruiz-Salmeron R. (2019). Cost-effective, safe, and personalized cell therapy for critical limb ischemia in type 2 diabetes mellitus. Front. Immunol..

[33] Klinkhammer B.M., Kramann R., Mallau M., Makowska A., van Roeyen C.R., Rong S., Buecher E.B., Boor P., Kovacova K., Zok S. (2014). Mesenchymal stem cells from rats with chronic kidney disease exhibit premature senescence and loss of regenerative potential. PLoS ONE.

[34] Lee S.J., Jung Y.H., Oh S.Y., Yun S.P., Han H.J. (2014). Melatonin enhances the human mesenchymal stem cells motility via melatonin receptor 2 coupling with galphaq in skin wound healing. J. Pineal Res..

[35] Hardeland R. (2017). Melatonin and the pathologies of weakened or dysregulated circadian oscillators. J. Pineal Res..

[36] Tang Y., Cai B., Yuan F., He X., Lin X., Wang J., Wang Y., Yang G.Y. (2014). Melatonin pretreatment improves the survival and function of transplanted mesenchymal stem cells after focal cerebral ischemia. Cell Transplant..

[37] Mortezaee K., Khanlarkhani N., Sabbaghziarani F., Nekoonam S., Majidpoor J., Hosseini A., Pasbakhsh P., Kashani I.R., Zendedel A. (2017). Preconditioning with melatonin improves therapeutic outcomes of bone marrow-derived mesenchymal stem cells in targeting liver fibrosis induced by ccl4. Cell Tissue Res..

[38] Langrzyk A., Nowak W.N., Stepniewski J., Jazwa A., Florczyk-Soluch U., Jozkowicz A., Dulak J. (2018). Critical view on mesenchymal stromal cells in regenerative medicine. Antioxid. Redox Signal..

[39] Munro D., Treberg J.R. (2017). A radical shift in perspective: Mitochondria as regulators of reactive oxygen species. J. Exp. Biol..

[40] Trub A.G., Hirschey M.D. (2018). Reactive acyl-coa species modify proteins and induce carbon stress. Trends Biochem. Sci..

[41] van der Reest J., Lilla S., Zheng L., Zanivan S., Gottlieb E. (2018). Proteome-wide analysis of cysteine oxidation reveals metabolic sensitivity to redox stress. Nat. Commun..

[42] Shutt T., Geoffrion M., Milne R., McBride H.M. (2012). The intracellular redox state is a core determinant of mitochondrial fusion. EMBO Rep..

[43] Bueno M., Lai Y.C., Romero Y., Brands J., St Croix C.M., Kamga C., Corey C., Herazo-Maya J.D., Sembrat J., Lee J.S. (2015). Pink1 deficiency impairs mitochondrial homeostasis and promotes lung fibrosis. J. Clin. Investig..

[44] Padman B.S., Bach M., Lucarelli G., Prescott M., Ramm G. (2013). The protonophore cccp interferes with lysosomal degradation of autophagic cargo in yeast and mammalian cells. Autophagy.

[45] Rahmani M., Nkwocha J., Hawkins E., Pei X., Parker R.E., Kmieciak M., Leverson J.D., Sampath D., Ferreira-Gonzalez A., Grant S. (2018). Cotargeting bcl-2 and pi3k induces bax-dependent mitochondrial apoptosis in aml cells. Cancer Res..

[46] Sinha K., Das J., Pal P.B., Sil P.C. (2013). Oxidative stress: The mitochondria-dependent and mitochondria-independent pathways of apoptosis. Arch. Toxicol..

[47] Takahashi S., Andreoletti G., Chen R., Munehira Y., Batra A., Afzal N.A., Beattie R.M., Bernstein J.A., Ennis S., Snyder M. (2017). De novo and rare mutations in the hspa1l heat shock gene associated with inflammatory bowel disease. Genome Med..

[48] Huusko J.M., Karjalainen M.K., Graham B.E., Zhang G., Farrow E.G., Miller N.A., Jacobsson B., Eidem H.R., Murray J.C., Bedell B. (2018). Whole exome sequencing reveals hspa1l as a genetic risk factor for spontaneous preterm birth. PLoS Genet..

[49] Jin S.M., Lazarou M., Wang C., Kane L.A., Narendra D.P., Youle R.J. (2010). Mitochondrial membrane potential regulates pink1 import and proteolytic destabilization by parl. J. Cell Biol..

[50] Lazarou M., Jin S.M., Kane L.A., Youle R.J. (2012). Role of pink1 binding to the tom complex and alternate intracellular membranes in recruitment and activation of the e3 ligase parkin. Dev. Cell.

[51] Nguyen T.N., Padman B.S., Lazarou M. (2016). Deciphering the molecular signals of pink1/parkin mitophagy. Trends Cell Biol..

[52] Han Y.S., Lee J.H., Yoon Y.M., Yun C.W., Noh H., Lee S.H. (2016). Hypoxia-induced expression of cellular prion protein improves the therapeutic potential of mesenchymal stem cells. Cell Death Dis..

