# Supplementary material for: Melatonin Enhances Mitophagy by Upregulating Expression of Heat Shock 70 kDa Protein 1L in Human Mesenchymal Stem Cells under Oxidative Stress
Source: Int J Mol Sci. 2019 Sep 13;20(18):4545. doi: 10.3390/ijms20184545 (PMC6769944; doi:10.3390/ijms20184545)
Supplement: Supplementary file 1 [file ijms-20-04545-s001.pdf]

Supplementary Figure

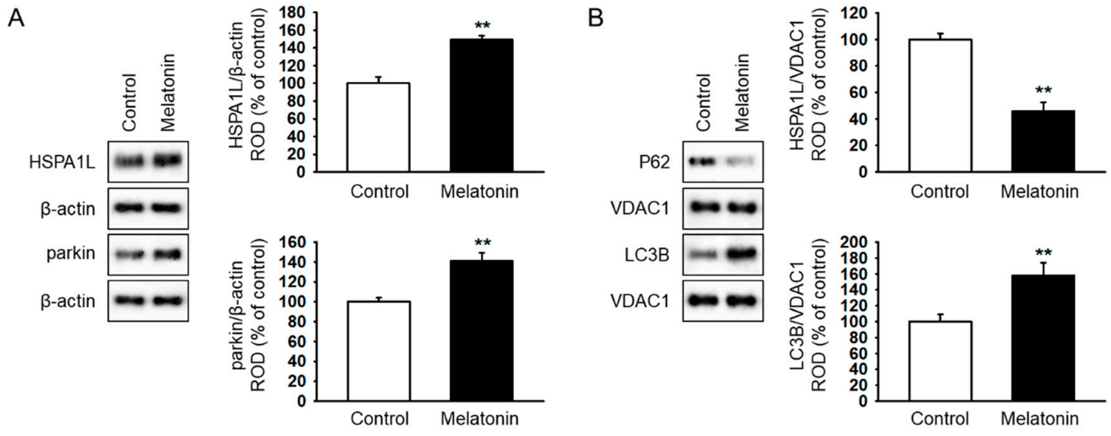

Supplementary Figure S1. Melatonin induces mitophagy by increasing the expression of HSPA1L and parkin. (A) Expression of HSPA1L and parkin in hMSCs treated with or without melatonin (1 μM) for 24 h. The expression levels of HSPA1L and parkin were normalized with respect to that of β-actin. Values represent the mean ± SEM. **$p < 0.01$ vs. untreated hMSCs. (B) Expression of P62 and LC3B in hMSCs treated with or without melatonin (1 μM) for 24 h. The expression levels of P62 and LC3B were normalized with respect to that of VDAC1. Values represent the mean ± SEM. **$p < 0.01$ vs. untreated hMSCs.

A

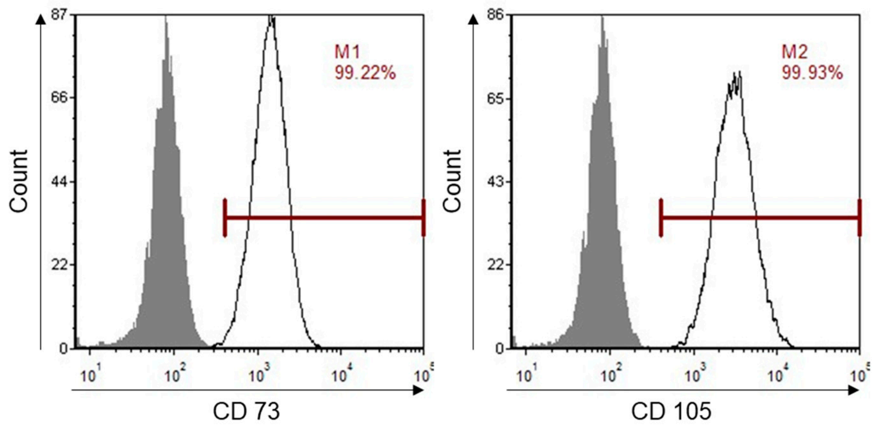

Supplementary Figure S2. Expression of hMSC surface markers. (A) MSC surface markers, including CD73, and CD105 were analyzed using flow cytometry. Gray lines indicate cell stained with isotype-matched IgG as a negative control. Black lines indicate cell stained with the indicated antibody.

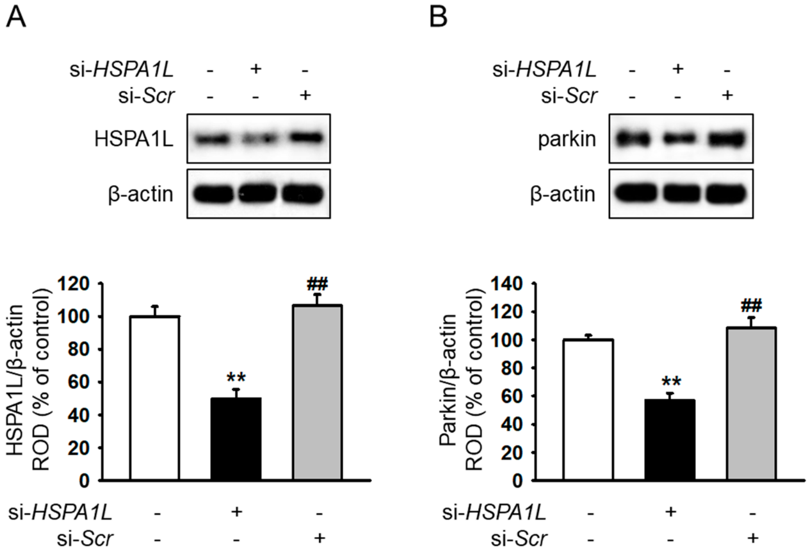

Supplementary Figure S3. Expression of HSPA1L and parkin in hMSCs after treatment with scrambled siRNA or si-*HSPA1L*. (A) Expression of HSPA1L in hMSCs treated with si-*HSPA1L* or scrambled siRNA (si-*Scr*). The expression level of HSPA1L was normalized with respect to that of β-actin. Values represent the mean ± SEM. **p < 0.01 vs. untreated hMSCs, and ##p < 0.01 vs. treated si-*HSPA1L*. (B) Expression of parkin in hMSCs treated with or without melatonin (1 μM) for 24 h. The expression level of parkin was normalized with respect to that of β-actin. Values represent the mean ± SEM. **p < 0.01 vs. untreated hMSCs, and ##p < 0.01 vs. treated si-*HSPA1L*.

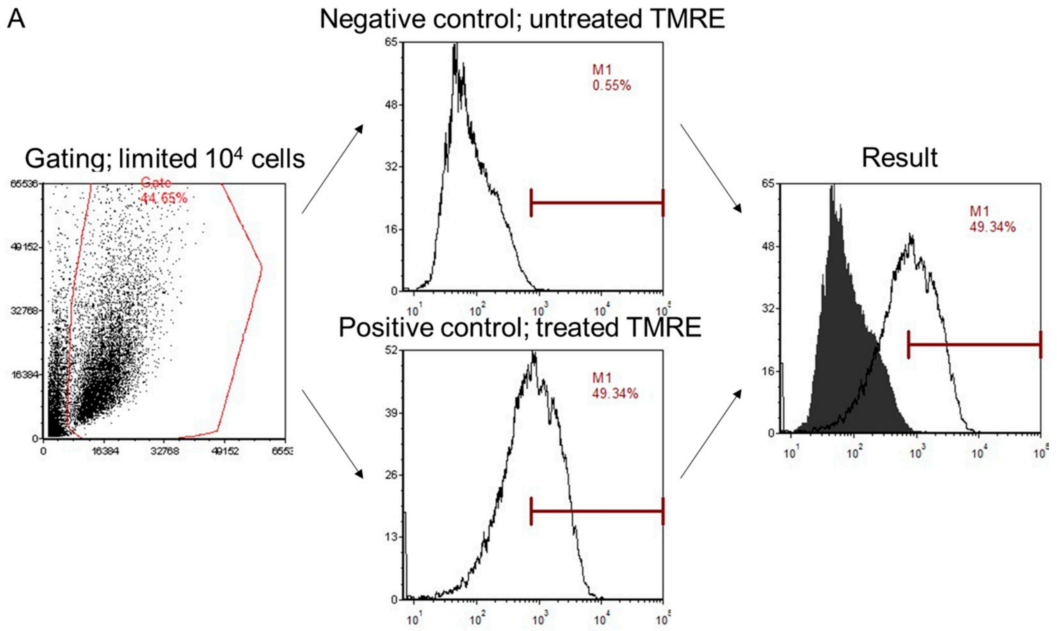

Supplementary Figure S4. FACS analyzed gating cells to distinguish the live cells from the total population. (A) Gray lines indicate the population of hMSCs untreated with TMRE as a negative control. Black lines indicate hMSCs stained with TMRE.

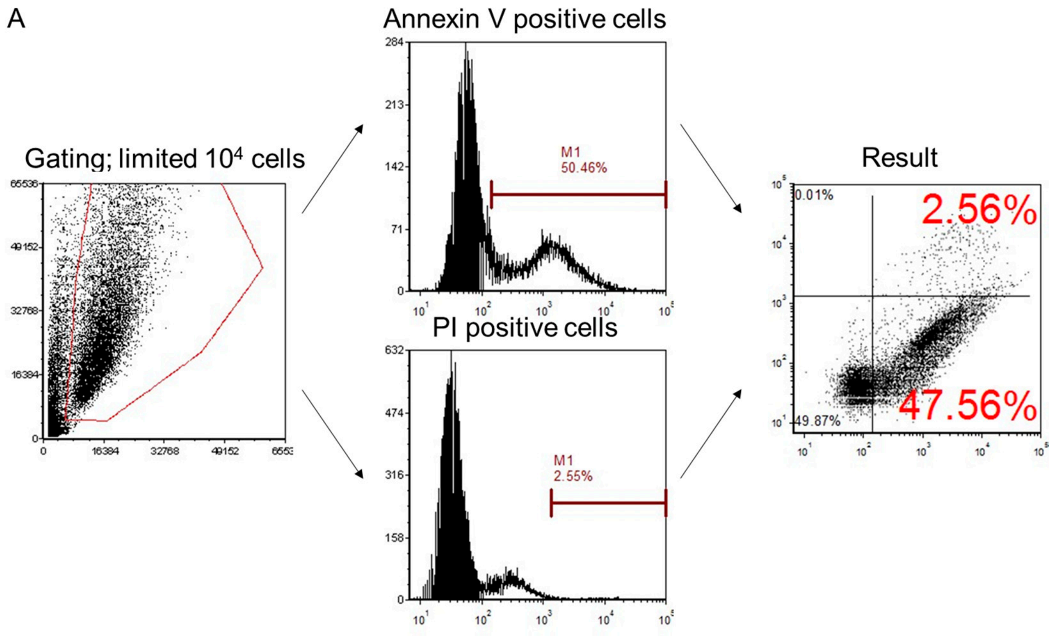

Supplementary Figure S5. Compensation of flow cytometry assay for Annexin V/PI staining. (A) Histograms for gating cells, Annexin V positive cells, PI positive cells, and Annexin/PI double positive cells.
